# Probing Field Cancerization in the Gastrointestinal Tract Using a Hybrid Raman and Partial Wave Spectroscopy Microscope

**DOI:** 10.1021/acs.analchem.5c00954

**Published:** 2025-06-11

**Authors:** Elena Kriukova, Mikhail Mazurenka, Sabrina Marcazzan, Markus Tschurtschenthaler, Gerwin Puppels, Sarah Glasl, Dieter Saur, Moritz Jesinghaus, Marialena Pouliou, Marios Agelopoulos, Apostolos Klinakis, Michael Quante, Jorge Ripoll, Vasilis Ntziachristos, Dimitris Gorpas

**Affiliations:** † Chair of Biological Imaging, Central Institute for Translational Cancer Research (TranslaTUM), School of Medicine and Health & School of Computation, Information and Technology, 9184 Technical University of Munich, Munich 81675, Germany; ‡ Institute of Biological and Medical Imaging, Bioengineering Center, Helmholtz Zentrum München, Neuherberg 85764, Germany; § Division of Translational Cancer Research, German Cancer Research Center (DKFZ) and German Cancer Consortium (DKTK), Heidelberg 69120, Germany; ∥ Chair of Translational Cancer Research and Institute of Experimental Cancer Therapy, Klinikum rechts der Isar, School of Medicine and Health, Technical University of Munich, Munich 81675, Germany; ⊥ Center for Translational Cancer Research (TranslaTUM), School of Medicine and Health, Technical University of Munich, Munich 81675, Germany; # RiverD International B.V., Marconistraat 16, AK, Rotterdam 3029, Netherlands; ¶ Institute of Pathology, Technical University of Munich, Munich 81675, Germany; ∇ Institute of Pathology, University Hospital Marburg, Marburg 35043, Germany; ○ Center for Basic Research, 89223 Biomedical Research Foundation of the Academy of Athens, Athens 115 27, Greece; ⧫ Klinik für Innere Medizin II, 14879 Universitätsklinikum Freiburg, Freiburg 79106, Germany; †† Department of Bioengineering and Aerospace Engineering, 16726 Universidad Carlos III de Madrid, Madrid 28005, Spain; ‡‡ Munich Institute of Biomedical Engineering (MIBE), Technical University of Munich,, Garching b, München 85748, Germany

## Abstract

Field cancerization (FC) refers to spatially distributed premalignant tissue changes that lead to the appearance of local malignancy, and its detection can improve cancer screening. In this work, we employ combined Raman and partial wave spectroscopy (RS-PWS) to detect FC in gastroesophageal (L2-IL1B) and intestinal (Villin-Cre, Apc^fl/wt^) tumor mouse models. Using a hybrid RS-PWS microscope, we acquire both molecular and morphological information from macroscopically normal tumor-adjacent tissue and investigate the individual and combined performance of each modality. For data analysis, we use partial least-squares discriminant analysis (PLS-DA). In the normal tissue of L2-IL1B mice, we demonstrate a statistically significant increase (*p* < 0.001) in Raman band intensities associated with free amino acids and a decrease in bands associated with lipids (*p* < 0.005) and carotenoids (*p* < 0.001) compared to healthy controls. Similarly, in the normal mucosa of Villin-Cre, Apc^fl/wt^ mice, the intensities of RS bands associated with amino acids increase significantly (*p* < 0.05) compared to controls, while the intensities of lipid-associated bands decrease significantly (*p* < 0.05). Transcriptomic profiling using RNA-sequencing analysis on these samples identified a significant correlation between gene expression and optical findings. Moreover, we demonstrate that combining RS and PWS data further improves the significance of our classification results. When macroscopically normal tumor-adjacent tissue is compared with tissue from healthy controls, we observe that PWS increases the *R* ^2^ of RS results by ∼9% in L2-IL1B mice and ∼5% in Villin-Cre, Apc^fl/wt^ mice. Combining molecular RS with structural PWS information enhances the ability to detect precancerous changes and provides insights into tissue alterations during cancer development.

## Graphical abstract

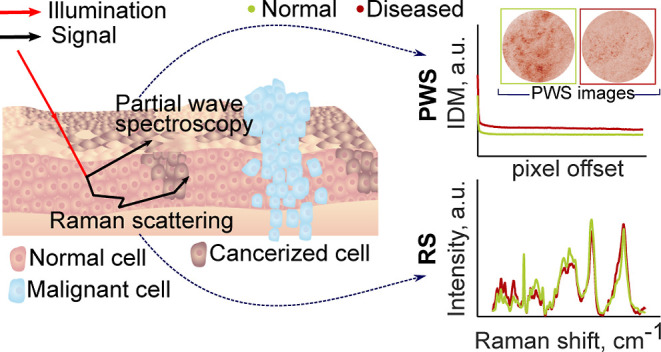

## Introduction

Field cancerization (FC), also called field carcinogenesis, refers to premalignant genetic and epigenetic alterations that cause subtle molecular and microstructural changes within tissues before a clear morphological appearance of a lesion. These alterations are indicative of cellular susceptibility to cancer progression.^1^ These changes may manifest across large parts of entire organs, i.e., appearing at much larger areas than the focal locations where lesions appear after disease progression. FC is associated with many types of cancer, including lung, esophagus, colon, skin, prostate, and bladder cancers.^1^ Because FC typically precedes the morphological signs of cancer lesions, detecting FC could allow for personalized preventive interventions and patient stratification early in the carcinogenesis process, possibly improving treatment efficacy. Early and reliable assessments of tissue changes associated with malignancy are especially important in the case of gastrointestinal (GI) cancers. Most GI cancers develop quite before the first symptoms and therefore are usually diagnosed at late and advanced stages, leading to high morbidity worldwide.^2^

Additionally, in gastroesophageal adenocarcinomas, which include esophageal adenocarcinoma (EAC) and cancer at the gastroesophageal junction (GEJ), chronic inflammation has been recognized as a key driver of cancer development.^3,4^ Individuals with gastroesophageal reflux disease (GERD)-associated inflammation do indeed have an increased risk for cancer due to promotion of stem cell expansion, genomic instability, and selection of mutated clones triggered by repeated epithelial injury.^5^ Barrett Esophagus (BE), a metaplastic response of the esophageal squamous epithelium to chronic exposure of acid and bile reflux, has long been viewed as a precursor to EAC.^4^ However, we have previously shown in our transgenic mouse model that IL1B-driven inflammation can initiate BE and EAC, particularly when combined with bile acids and nitrosamines.^6^ Therefore, the visible metaplasia alone may not be sufficient to predict cancer risk.^5^ Additional markers including stem cell activity, genomic instability, and inflammatory markers would give further insights into FC and cancer progression, but no standardized approaches or biomarkers to identify such changes have been reported yet.^1,7^

Currently, pathology evaluation is the most prevalent way to determine the cancer risk in conditions related to FC,^1^ using procedures such as histological examination of hematoxylin and eosin (H&E)-stained biopsy samples.^8^ In GI cancers, biopsies are performed using traditional white light endoscopy (WLE),^2^ which is known to have high miss rates (dysplasia miss rates of around 57% in the esophagus and adenoma detection miss rates of up to 22% in the colon).^9^ The sampling errors, subjectivity, and substantial cost associated with these pathological procedures also limit their ability to assess precancerous conditions. Furthermore, FC can be histologically indistinguishable from healthy tissue and thus cannot be diagnosed solely by pathology.^1^

To date, FC in the GI tract has been extensively studied with various scattering-based techniques enabling the investigation of different structural facets of field carcinogenesis^10,11^ even before changes become visible to microscopy. Polarization-gated spectroscopy (PGS), low-coherence enhanced backscattering spectroscopy (LEBS), and partial-wave spectroscopic microscopy (PWS) have been employed as means for FC detection. PGS has been used to quantify rectal hemoglobin concentration ([Hb]) in mucosal blood content,^12^ where early increase in blood supply (EIBS) has been observed to precede tumor formation. However, correlations between critical tissue changes and microvascular abnormalities still need to be investigated, as well as factors that determine an individual’s critical threshold of blood supply, a necessary parameter to predict the risk of developing a lesion.^13^

LEBS has been used to detect the disorganization of macromolecules (e.g., chromatin and extracellular collagen), which are related to epithelial FC. LEBS was employed to assess a number of tissue features associated with colorectal cancer.^14^ Analysis of data from 619 patients detected FC with 88% sensitivity and 72% specificity. Nevertheless, measurements from a large number of patients (*n* = 93) had to be excluded due to unreliable measurements.

In studies of colorectal^15^ and esophageal^16^ FC, PWS^17^ has previously been used to detect mass density variations of cellular building blocks (proteins, RNA, and DNA) based on a single parameter that indicates the disorder strength (*L*_d_) of patients’ cells and compartments of those cells (e.g., the cell nucleus). In both studies, the cells were harvested from patients undergoing endoscopic surveillance and therapeutic interventions, and the PWS imaging was performed on the cells obtained with a cytology brush.

However, PWS has only been applied in vitro, and its performance was reported based only on *L*_d_ with relatively high standard deviation.^18^ On the other hand, multidiameter single fiber reflectance spectroscopy (MDSFR) is an optical technique that has been used for in vivo FC detection^19,20^ in patient cohorts (*N* = 33–48). However, the MDSFR specificity in detecting FC has been proven similar to or smaller than PWS^16^ and LEBS.^21^

Raman spectroscopy (RS) has also previously been considered for the detection of FC or other premalignant conditions such as dysplastic Barrett’s esophagus (BE), colon polyps, or oral cancers.^7,22–24^ RS detects biomolecular signatures which are indicative of progression toward malignancy (e.g., changes in the concentrations of proteins, DNA, lipids, glycogen, etc.) in various tissue types.^24^ Several clinical RS studies were also focused on identifying RS signatures in malignant lesions of the GI tract.^22,24^ While some studies have included premalignant lesions,^23,25^ they have not specifically examined FC effects or differentiated healthy areas from normal-appearing areas close to early lesions. For example, normal and hyperplastic tissues were treated as one benign group in a classification model for the in vivo diagnosis of colorectal polyps.^25^ Another study reported minor RS differences between normal tissue and hyperplastic polyps and could not distinguish between hyperplastic polyps and adenomas.^23^

In addition to the clinical trials, a preclinical study^26^ investigated the adenoma-carcinoma sequence in a mouse model of colon carcinogenesis using RS. It was shown, based on the systematic evaluation of Raman spectra recorded from tissue cryosections, that the altered tissue (i.e., hyperplastic tissue) was not distinguishable from healthy tissue across all four stages of the adenoma-carcinoma sequence. The findings suggest that the differences between normal and adenoma tissue are greater than between normal tissue and hyperplasia.^26^ However, to date, RS has not been used to explicitly study FC effects in the GI tract or to characterize the biochemical changes responsible for cancer development before these changes can be distinguished in H&E-stained sections by a trained histopathologist. Therefore, current methods for detecting both structural and chemical FC biomarkers are insufficient to obtain a comprehensive picture of FC in tissue.

In this study, we aimed to investigate the relative performance of two optical modalities, RS and PWS, for detecting structural and molecular changes associated with FC and examined if their combination can improve the detection accuracy. For this comparison, we employed a custom-developed hybrid RS-PWS microscope that we described in depth elsewhere.^27^ Unlike our previous work, which established the system and provided proof-of-principle measurements, herein we interrogated, for the first time, field cancerization effects in mouse models of gastroesophageal (L2-IL1B, here referred to as IL1B)^28^ and intestinal (Villin-Cre, Apc^fl/wt^, here referred to as Apc)^29^ cancer. Our analysis assessed both the individual and combined performance of each modality by evaluating biomolecular and morphological changes observed in the spectroscopic data.

We assessed data collected from forestomach tissue adjacent to precancerous squamocolumnar junction (SCJ) tissue in IL1B mice compared to healthy controls. We observed a statistically significant decrease in the RS intensities of bands associated with lipids and carotenoids and a statistically significant increase in the intensities of bands assigned to free amino acids. Moreover, we also examined both precancerous adenomas and macroscopically normal tumor-adjacent mucosa from intestinal tumor mouse models (Apc) compared to healthy controls. Here, the intensity of the Raman spectral bands associated with amino acids increased significantly in Apc mice, while the intensity of a band associated with lipids decreased. To support the RS-PWS findings, we also conducted RNA sequencing (RNA-seq) on all tissue samples.

Finally, by applying multivariate analysis, specifically partial least-squares discriminant analysis (PLS-DA), we demonstrated that the combined RS and PWS data achieved higher sensitivity for FC detection in tissues than either modality alone.

## Materials and Methods

### Animal Models and Sample Preparation

#### Gastroesophageal Tumor Mouse Model (L2-IL1B, Ref as IL1B)

The L2-IL1B mouse model aims to reproduce the sequence of BE and adenocarcinoma development in humans. L2-IL1B mice were generated as previously reported^28^ and backcrossed with C57BL6/J mice. L2-IL1B mice were genotyped at 21 days and fed with water and standard food ad libitum (ssniff, Germany).

Ten- to fifteen-month old male/female L2-IL1B mice (*n* = 26) and age-matched C57BL6/J wild-type (WT) (*n* = 10, Charles River) mice were sacrificed with an overdose of isoflurane. Immediately after the mice were sacrificed, the stomachs were opened along the large curvature and washed with phosphate buffered saline (PBS). The cardia and esophagi were then opened while the tissue was kept on a Petri dish with ice and distended. A snapshot picture (via a dissection microscope) of the whole stomach and whole esophagus (distal and proximal) was taken for each sample, and tumor and normal-appearing areas were identified. Finally, each stomach was divided into two equal parts under a dissection microscope. One half was immediately fixed in 4% paraformaldehyde (PFA) overnight for histopathology analysis, and the other half was used for optical measurements and RNA-seq analysis (Figure 1a). Tissue pieces (typically 2 mm × 2 mm, minimum 1 mm × 1 mm) were then cut for RNA isolation from the forestomach, SCJ/cardia region, and stomach. Samples for RNA-seq analysis were immediately snap-frozen in liquid nitrogen and stored at −80 °C.

**Figure 1:**
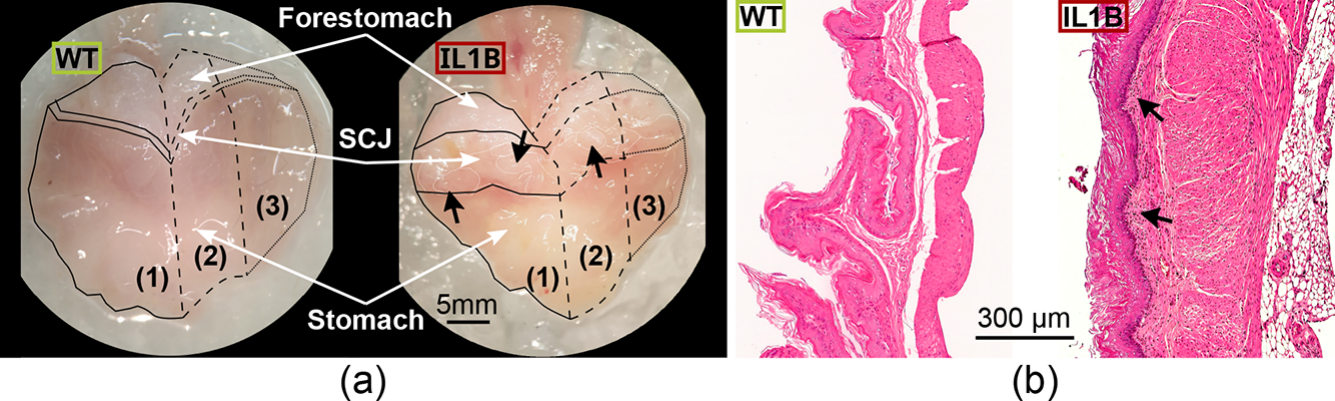
Exemplary microscope and H&E pictures from mouse stomach samples. (a) Dissection microscope photos of tissue samples from the whole stomach (left—wild type (WT); right—IL1B). SCJ—squamocolumnar junction. Mouse stomachs are divided into two equal parts with one half fixed in 4% PFA for histology (1) and the other half divided between optical measurements (2) and RNA sequencing analysis (RNA-seq) (3). (b) Representative images of H&E-stained forestomach samples of both WT and IL1B. The black arrows point at areas with an increased influx of inflammatory cells.

#### Intestinal Tumorigenesis Mouse Model (Villin-Cre, Apc^fl/wt^, Ref as Apc)

Intestinal epithelial cell-specific deletion of one Apc allele was generated by means of the Villin-Cre/loxP system.^30^ The floxed Apc^fl^ allele has been previously described^29^ and was crossed to Villin-Cre mice. All mice were from a C57Bl/6 background. Ear biopsy genomic DNA was used for genotyping.

Male and female 7–18 month-old Villin-Cre, Apc^fl/wt^ mice (*n* = 7) were euthanized by isoflurane and subsequent cervical dislocation when they reached humane end point criteria. WT littermate animals containing only Cre recombinase or no Cre recombinase served as age-matched controls (*n* = 9). Parts of the small intestines were collected following a similar sample preparation protocol as for the gastroesophageal tumor mouse model. The collected parts were opened, washed with PBS, and divided into three parts under a dissection microscope for measurements with our multimodal system, histology, and RNA-seq analysis. The samples were divided into normal tissue samples (Apc-NT) and tumor tissue samples (Apc-T), which were set aside for optical measurements. Another section containing both tumor and normal tissue was fixed in 4% PFA for histology. Apc-NT and Apc-T samples were also collected for the RNA-seq analysis. Tumors were counted in each part of the Apc mice intestines (duodenum, jejunum, ileum, and colon) to assess the tumor burden.

All animal studies were conducted in compliance with European guidelines for the care and use of laboratory animals and were approved by the Institutional Animal Care and Use Committees (IACUC) of the local authorities of Technische Universität München and the Regierung von Oberbayern (animal protocol numbers: ROB-55.2–2532.Vet_02–17–79; ROB-55.2–2532.Vet_02 −15–29; and ROB-55.2–2532.Vet_02–20–69). Animals were housed under specific pathogen-free conditions (SPF) in a dedicated facility, with a light–dark cycle of 12:12 h, an air humidity between 45 and 65%, and a temperature between 20 and 24 °C.

### Histopathology Sample Preparation

The samples for histopathological examination were fixed in 4% PFA and further processed for paraffin embedding. The cut sections were stained with hematoxylin and eosin (H&E).

### RNA Sequencing Sample Preparation

The tissue samples for RNA analysis were snap-frozen in individual Eppendorf tubes in liquid nitrogen immediately after collection and stored at −80 °C. The frozen tissue samples were transferred to separate Precellys tubes (Bertin Technologies), and Trizol reagent (1 mL) was added. The tubes were then transported on ice to a Precellys Evolution homogenizer (Bertin Technologies). The samples were homogenized twice at 6500 rpm, each for 20 s. For samples not completely homogenized, the cycle was repeated. The supernatant of the homogenized content was stored in Trizol at −80 °C until RNA isolation.

RNA-seq was conducted to assess the expression of genes in mice with gastroesophageal and intestinal tumors and in their respective control groups. An initial step of RNA extraction, purification, and isolation was followed by the preparation of next-generation sequencing (NGS)-compatible multiplexed libraries.^31^ Finally, next-generation sequencing was performed with the use of the NovaSeq 600 Illumina system.^31^

### System and Data Acquisition

The hybrid RS and PWS microscope used in this study has been developed by our group and is detailed elsewhere.^27^ Briefly, the microscope body was assembled from Cerna Microscope Components (Thorlabs) and incorporates an XY stage for sample scanning and a Z stage for focus adjustment. A multiobjective nosepiece accommodates a Raman objective (RiverD) and low-NA PWS objective (RMS20X, Thorlabs) that are exchanged depending on the type of measurements. A move-in mirror allows for rapid switching between the optical paths for RS and PWS.

Each tissue sample was measured with both modalities at several fields of view (FOVs). For the gastroesophageal tumor model, the FOVs were chosen at the forestomachs of the excised stomach samples (Figure 1a). For the intestinal tumor mouse model, one FOV was measured per tissue sample, while for the control mice’s intestines, several FOVs were measured per tissue sample. LabVIEW-based software was designed and developed to control the RS-PWS microscope and acquire all data. The samples for optical measurements were prepared according to the protocol reported in a previous study of our group.^27^

#### Raman Data Acquisition

Raman spectra for each field of view (FOV) were recorded in a 10 × 10 pixel grid pattern with a 50 μm spacing between pixels, yielding 100 spectra per FOV. These measurements were made using a high-performance Raman module (RiverD International, Rotterdam, The Netherlands) integrated into the microscope body.^27^ Integration times were set to 10 and 2 s per spectrum for the high wavenumber region (HWVN) and the fingerprint region (FP), respectively (excitation wavelengths: 671 and 785 nm).

#### PWS Data Acquisition

A stack of 151 images per each FOV was recorded with a CCD camera (Grasshopper3 GS3-U3–28S5M, Point Gray) from backscattered light (i.e., white LED) focused through the low NA objective and spectrally filtered using a liquid crystal tunable filter (KURIOS-WB1/M, Thorlabs) to obtain a final PWS image using the algorithm described in ref 32. Each FOV was recorded at wavelengths ranging from 550 to 700 nm with 1 nm steps. Before each measurement, the instrument response function (IRF) was also measured by acquiring an image stack of the empty slide.

### Data Preprocessing

#### RS Data

Before each measurement session, we performed a calibration sequence on the Raman module, using built-in software, as previously reported.^27^ For further processing, the following spectral regions were used: (i) fingerprint (FP) region: 800–1800 cm^–1^; (ii) high wavenumber (HWVN) region: 2800–3050 cm^–1^. The FP region was limited to those wavenumbers, as no signal changes were observed below 800 cm^–1^, as also shown in previous studies.^23,25,33,34^ On the other hand, the HWNV region was limited below 3050 cm^–1^, as all tissue samples were measured ex vivo, and the water content most probably would decrease with the duration of the measurement procedure, which, in turn, would bias the analysis. Moreover, the PBS drops used for sample preparation would also interfere with the measurements at bands higher than 3050 cm^–1^. All acquired spectra were smoothed with a first-order Savitsky–Golay filter and corrected for fluorescence background by means of an improved modified multipolynomial (iModPoly) fitting function.^35^ A fifth-order polynomial was used for fitting the broad autofluorescence background of the FP spectra, and a third-order polynomial was optimal for the HWVN spectra.

To make spectra comparable and to scale differences between them, vector normalization was applied for each set of spectra recorded per FOV. Briefly, the “norm” of each spectrum was first calculated as the square root of the sum of the squared intensities of the spectrum. Then, each of the RS intensities corresponding to a Raman shift was divided by the “norm” to obtain the normalized spectrum.^36^ For each spectral data set with distinct outliers, density-based spatial clustering of applications with noise (DBSCAN) classification was applied as an additional step to obtain the final spectral data set (i.e., without outliers and noise). For intestinal samples, spectra in the HWVN region were of insufficient quality and were not used in the final analysis. We hypothesized that this insufficient quality was due to the dehydration of the thin intestinal tissue samples.

#### PWS Data

PWS images of tissue samples were evaluated using the gray-level co-occurrence matrix (GLCM).^37^ From each GLCM, the inverse difference moment (IDM) textural feature was derived by using standard MATLAB (R2019b, MathWorks) functions. This IDM parameter was used to describe the texture of images and estimate slight variations in the sample surface structure.

### Multivariate and Statistical Analysis of All Recorded RS-PWS Microscope Data

The unpaired nonparametric Mann–Whitney *U* test was performed to check for statistically significant differences and calculate the p-values of the RS data. A partial least-squares discrimination analysis (PLS-DA) classification method with *k*-fold (*k* = 4) cross-validation (CV) was employed in MATLAB^27,38^ to discriminate WT or control tissues from macroscopically normal tissue areas adjacent to premalignant tumors (IL1B and Apc-NT groups). The results were compared for three data sets: (i) IDM textural feature values from PWS data, (ii) RS data, and (iii) concatenated and standardized PWS and RS data, after the z-score function was applied to place all data sets on the same scale. A one-sigma heuristic approach^39^ was used for the optimal number of components of each data set.^27^

### Data Analysis for RNA Sequencing

#### Bioinformatics Analysis

To support the biomarkers identified by RS and PWS measurements, we performed RNA-seq to measure, quantify, and classify the gene expression changes accompanying the development of the phenotypes of interest. The primary RNA-seq data from a total of 104 samples, including both mouse models, were analyzed using the Galaxy online data analysis platform.^40^ The first step of the analysis utilized raw sequence data and assays to assess the quality of the sequencing reads through the application of FastQC (Galaxy Version 0.72+galaxy1). Next, adapter trimming and removal of reads characterized by a quality score lower than 20 were performed by the application of Trim Galore software, followed by the alignment/mapping of reads on the mm9 reference mouse genome, according to HISAT2 alignment tool specifications. Default parameters were applied (“single-end”, “stranded”, and “reverse” options; Galaxy Version 2.2.1+galaxy0).^41^

For the calculation of sequencing reads that are mapped across genes, the HTSeq-count algorithm was utilized (Galaxy Version 0.9.1)^42^ in union mode, with “stranded” and “reverse” options. Signal intensity was quantified by using the bamCoverage tool,^43^ which generates coverage bigWig files from BAM files. The RSeQC package was used for RNA quality control.^44^ See Supporting Information Tables S1–S6 for details.

Differential gene expression was determined using the DESeq2 algorithm (Galaxy Version 2.11.40.6+galaxy2),^45^ which is based on a model using the negative binomial distribution. Gene ontology classification (GO) and Kyoto Encyclopedia of Genes and Genomes (KEGG) pathway analyses of differentially expressed genes (DEGs) were performed using Database for Annotation, Visualization and Integrated Discovery (DAVID) software.^46^

The analyses for both tumor models were performed as follows: tissue pairs were compared with one another (i.e., WT forestomachs vs IL1B forestomachs, control intestine vs Apc-NT intestine, control intestine vs Apc-T intestine, and Apc-NT intestine vs Apc-T intestine). Each pairwise comparison yielded differentially expressed genes (DEGs) for all of the pairs. These genes were consequently grouped based on the cellular processes they are involved in (GO analysis) and pathways (KEGG).

## Results

### Gastroesophageal Tumor Mouse Model

Figure 1a shows microscope photographs of stomachs from a control (WT) mouse and a diseased (IL1B) mouse. Compared with the much thinner SCJ observed in the WT sample, the IL1B sample has an enlarged SCJ with nodules (black arrows), features that are linked to neoplastic processes. Optical measurements, sections for histology, and samples for RNA-Seq were also obtained from the forestomachs of IL1B (*n* = 26) and WT mice (*n* = 10). Forestomachs were selected for analysis because they contain the same squamous epithelium as the esophagus and are adjacent to the SCJ. For further analyses, the forestomachs of WT mice were considered “healthy”, while the forestomachs of IL1B mice, without visible nodules, were considered “macroscopically normal”.

Figure 1b displays an H&E-stained section from an 11 month-old IL1B mouse compared to a section from a WT mouse. The IL1B section exhibits an increased influx of inflammatory cells (black arrows) and signs of epithelial cell turnover (mitosis and increased keratinization). This is because the forestomach tissue of IL1B mice is targeted by IL1B overexpression as well as adjacent dysplastic SCJ tissue.^6^

Figure 2a depicts representative PWS images obtained from WT and IL1B mouse forestomachs. Figure 2b shows the corresponding inverse difference moment (IDM) textural feature derived from gray-level co-occurrence matrices (GLCMs) calculated for PWS fields of view (FOVs) from each group (red, IL1B, 26 FOVs; green, WT, 10 FOVs) with means and standard deviations indicated. IDM is plotted as a function of pixel offset, which is a function of the distance between pixels in an image and the direction of their offset from each other. The standard deviation (shaded area) of the resulting IDM textural feature values is relatively high for both groups. Mean IDM textural feature values also appear higher for the IL1B group. Although this difference was not statistically significant, the principal component analysis (PCA) score plots (see Supporting Information, Figure S1a) confirm both the difference between the groups and the high standard deviations of the IDM textural feature.^16,19,20^

**Figure 2:**
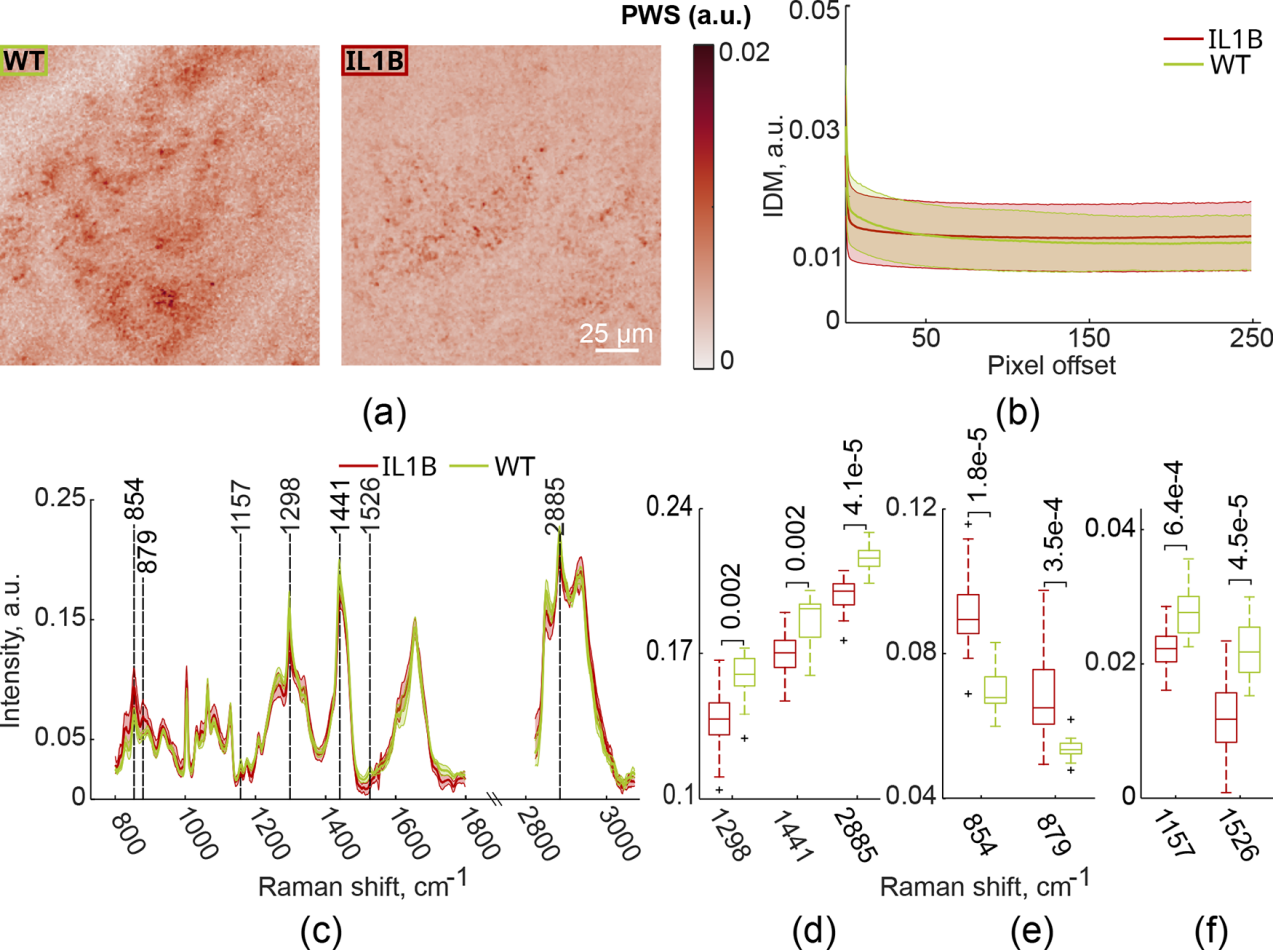
Partial wave spectroscopy (PWS) and Raman spectroscopy (RS) measurements from forestomach tissue samples. (a) Exemplary PWS images. (b) Distribution plots of the inverse difference moment (IDM) textural feature derived from gray-level co-occurrence matrices (GLCMs) calculated for each PWS image of forestomach tissue samples. (c) Raman spectra. (d–f) Boxplots of Raman spectra intensities which show significant differences between IL1B mice and controls (*p*-values are indicated within the bar graph; unpaired nonparametric Mann–Whitney *U* test). Red—IL1B mouse model; green—wild type mouse model (WT).

On the other hand, Raman spectra averaged over 26 forestomach FOVs from IL1B mice (red) and over 10 FOVs from WT mice (green) are shown in Figure 2c, with shaded areas corresponding to their standard deviations. The average Raman spectrum obtained from the WT group fluctuated less than that of the IL1B group tissues. To assess the statistical significance of differences between the spectra of IL1B and control (WT) groups, an unpaired nonparametric Mann–Whitney *U* test was performed. Application of the Mann–Whitney *U* test revealed a statistically significant decrease (*p* < 0.005) in spectral peaks of the IL1B group at wavelengths 1298, 1441, and 2885 cm^–1^, which correspond to lipids and fatty acids (Figure 2d), as well as at 1157 and 1526 cm^–1^, corresponding to carotenoids (Figure 2e). A significant increase (*p* < 0.001) of IL1B spectral intensities at 854 and 879 cm^–1^ is attributed to an increase in proteins and amino acids (i.e., hydroxyproline, tryptophan, and tyrosine) (Figure 2f). These results indicate that there are significant changes in the proportions of biomolecules in the forestomach tissues of IL1B mice (in agreement with the scatter plot of the first two PC scores for the RS data; Figure S1b).

To assign functional relevance to the differences observed in gene expression, we performed gene ontology (GO) and pathway analysis on differentially expressed genes (DEGs; see Materials and Methods) between WT and IL1B mice (see Supporting Information Tables S1 and S3). Results of these analyses indicate extensive expression changes in genes associated with inflammation. However, several other processes were also found to be affected, including tumorigenic cell signaling (signal transduction and others), epithelial organization and differentiation (keratinization, keratinocyte and epidermis development), wound healing, and others. Some of these processes can be linked to the RS statistical analysis results. Specifically, the “retinol metabolism” process found in the downregulated genes can be matched to the decrease of the 1526 cm^–1^ and 1157 cm^–1^ carotenoid peaks in the IL1B group (Figure 2f). Similarly, the “lipid metabolic process” and “fatty acid metabolic process”, which were found at the top of the GO list of downregulated genes, can be correlated to the decreased intensity of peaks associated with lipids and fatty acids in the IL1B group (1298, 1441, and 2885 cm^–1^) (Figure 2d). Thus, these Raman fingerprints can be considered as field cancerization markers, specific for esophageal cancer.

### Intestinal Tumor Mouse Model

Figure 3a displays microscope images of small intestine tissue samples prior to sectioning. The intestine of an Apc mouse has tumors that can be identified macroscopically. Optical measurements, sections for histology, and samples for RNA–Seq were obtained from precancerous adenoma tumors (Apc-T, *n* = 7), from adjacent “normal” mucosa (Apc-NT, *n* = 7), and from the small intestinal mucosa of control mice (n = 9). As before, the intestine samples from control mice were referred to as “healthy”, and intestinal tissue samples collected from the area adjacent to tumors in Apc mice were considered “macroscopically normal”.

**Figure 3:**
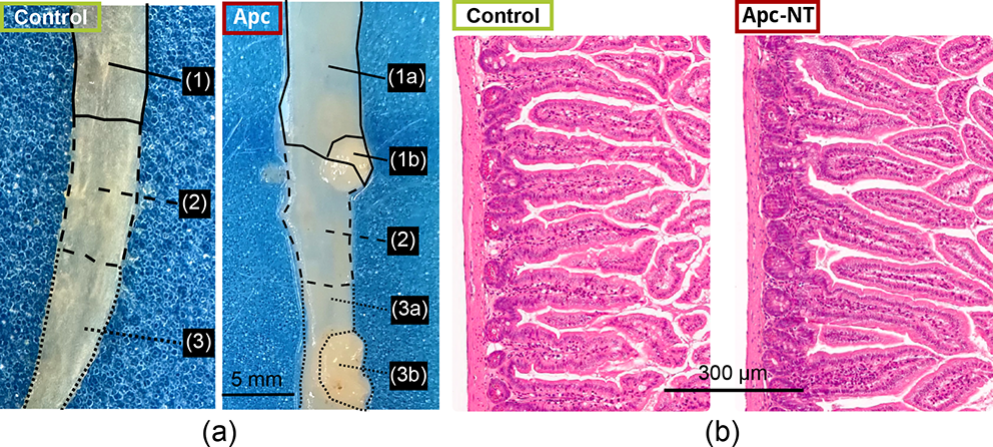
Exemplary microscope and H&E pictures from mouse intestinal samples. (a) Dissection microscope photos of freshly excised tissue samples (left—control; right—Apc) taken from the small intestine. After photos were taken, the intestine was divided into multiple parts: a normal tissue (Apc-NT) sample (1a) and a tumor tissue sample (Apc-T, 1b) taken for optical measurement, a tissue sample containing both normal and tumor tissues (2) taken for histology after fixation in 4% PFA, and Apc-NT (3a) and Apc-T (3b) samples collected for RNA sequencing (RNA-seq). (b) Representative H&E-stained images of the small intestine epithelium from control and Apc-NT samples.

Figure 3b shows representative images from histopathological analyses conducted using healthy and macroscopically normal intestinal samples with the histomorphological tissue structure of the Apc-NT sample not appearing significantly different from that of the control sample. Representative PWS images from the control and Apc-NT groups are shown in Figure 4a. There were similarly no observable differences in PWS images from both groups; however, the mean value of the IDM textural feature derived from GLCMs calculated for PWS images of the Apc-NT group (*n* = 7, 12 FOVs) is increased compared to the same value in the control group (*n* = 9, 26 FOVs) (Figure 4b). Moreover, the significant standard deviation (shaded area) of the IDM textural feature in the Apc-NT group suggests heterogeneity in the tissue morphology (confirmed in the score plot, Figure S2a). This heterogeneity is, however, expected as subtle structural changes may or may not have occurred uniformly in all cells within each FOV used for the IDM quantification. Nevertheless, the results of the PWS measurements imply morphological changes in intestinal mucosa adjacent to adenomas in Apc mice in comparison to healthy mucosa.

**Figure 4:**
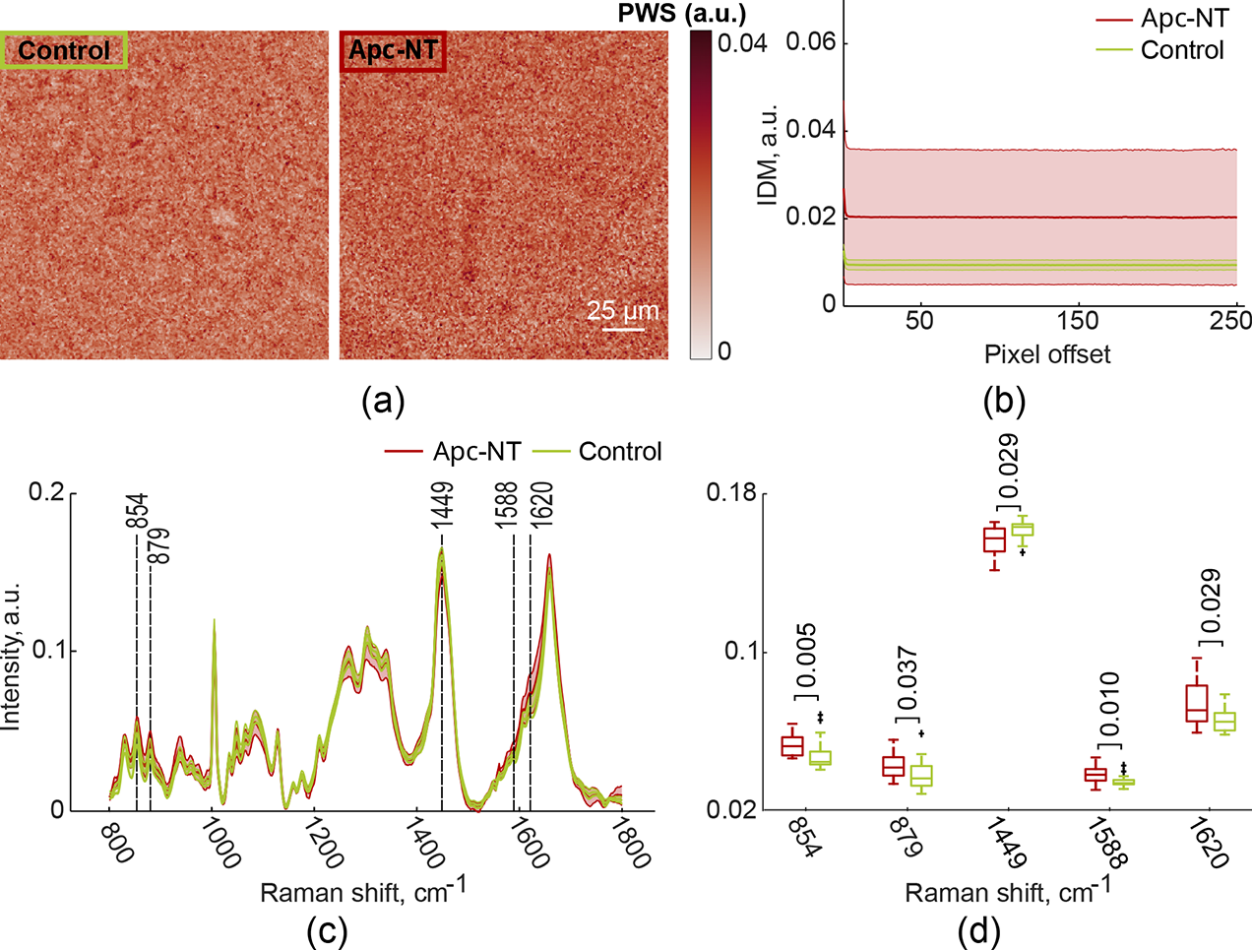
Partial wave spectroscopy (PWS) and Raman spectroscopy (RS) measurements from intestinal fields of view (FOVs). (a) Exemplary PWS images. (b) Distribution plot of the inverse difference moment (IDM) textural feature derived from gray-level co-occurrence matrices (GLCMs) calculated for each PWS image of intestine tissue samples. (c) Raman spectra. (d) Boxplots of Raman spectra intensities which show significant differences between Apc mice and controls (*p*-values indicated within the bar graph; unpaired nonparametric Mann–Whitney *U* test). Red—Apc-NT mouse model; green—control.

The Raman spectra averaged over 12 ‘normal’ (Apc-NT) FOVs from 7 Apc mice (red) and over 26 FOVs from 9 control mice (green) are shown in Figure 4c, with shaded areas representing standard deviations. A Mann–Whitney *U* test revealed a statistically significant increase (*p* < 0.05) in the intensity of spectral bands corresponding to free amino acids (e.g., proline, hydroxyproline, tyrosine, guanine, adenine, cytosine, and porphyrin) in the Apc-NT group compared to controls (wavelengths 854, 879, 1588, and 1620 cm^–1^, Figure 4d). A significant decrease (*p* < 0.05) of band intensities in the Apc-NT group is also observed at 1449 cm^–1^ (C–H vibrations, proteins/lipids) (Figure 4d). The lipid-to-protein ratio being higher in the control group is similar to our observations in IL1B mice (Figure 2d–f), suggesting that Raman spectroscopy can potentially diagnose FC in the GI tract based on tissue biomolecular signatures.

Similar to the gastroesophageal tumor model, RNA-seq followed by GO and pathway analysis on DEGs was performed for control and Apc-NT samples (see Tables S2 and S4). Some of the identified processes may be related to the statistically significant differences observed in the RS measurements. Interestingly, pathways and functions such as protein digestion and absorption, collagen formation, and collagen degradation (Table S4) can be associated with free amino acid Raman signatures (854, 879, 1588, and 1620 cm^–1^, Figure 4d). In addition, the “lipid metabolic process” and “lipid localization process” found in the GO list of downregulated genes can be correlated to the decrease in the lipid-associated peak (1449 cm^–1^, Figure 4d). The PWS and RS data from Apc-NT and control groups were also compared to the Apc-T group to observe alterations related to the development of precancerous adenomas (Supporting Information, Figures S3 and S4).

### PLS-DA Classification Results

Figures 5 and 6 show the results of the PLS-DA algorithm for PWS and RS data sets collected from both gastroesophageal (IL1B) and intestinal (Apc) tumor models, respectively. The classification results were computed with the optimal number of components chosen per data set using the one-sigma strategy (i.e., selection of the fewest components that are less than one standard error away from the overall best result).^39^ To quantify the performance of the PLS-DA algorithm, R^2^ and norm of residuals (||r||) were evaluated for each data set. For both tumor models, RS performs better than PWS when utilized alone. However, in the control group of intestinal samples, the fitted response of PWS data points showed less variation than the corresponding fitted response of RS data points (Figure 6a–b). Figures 5c and 6c illustrate that classification results are improved when the data from both modalities are used, with an *R* ^2^ = 0.819 and ||*r*|| = 1.143 for the gastroesophageal and an *R* ^2^ = 0.806 and ||*r*|| = 1.262 for the intestinal tumor models.

**Figure 5:**
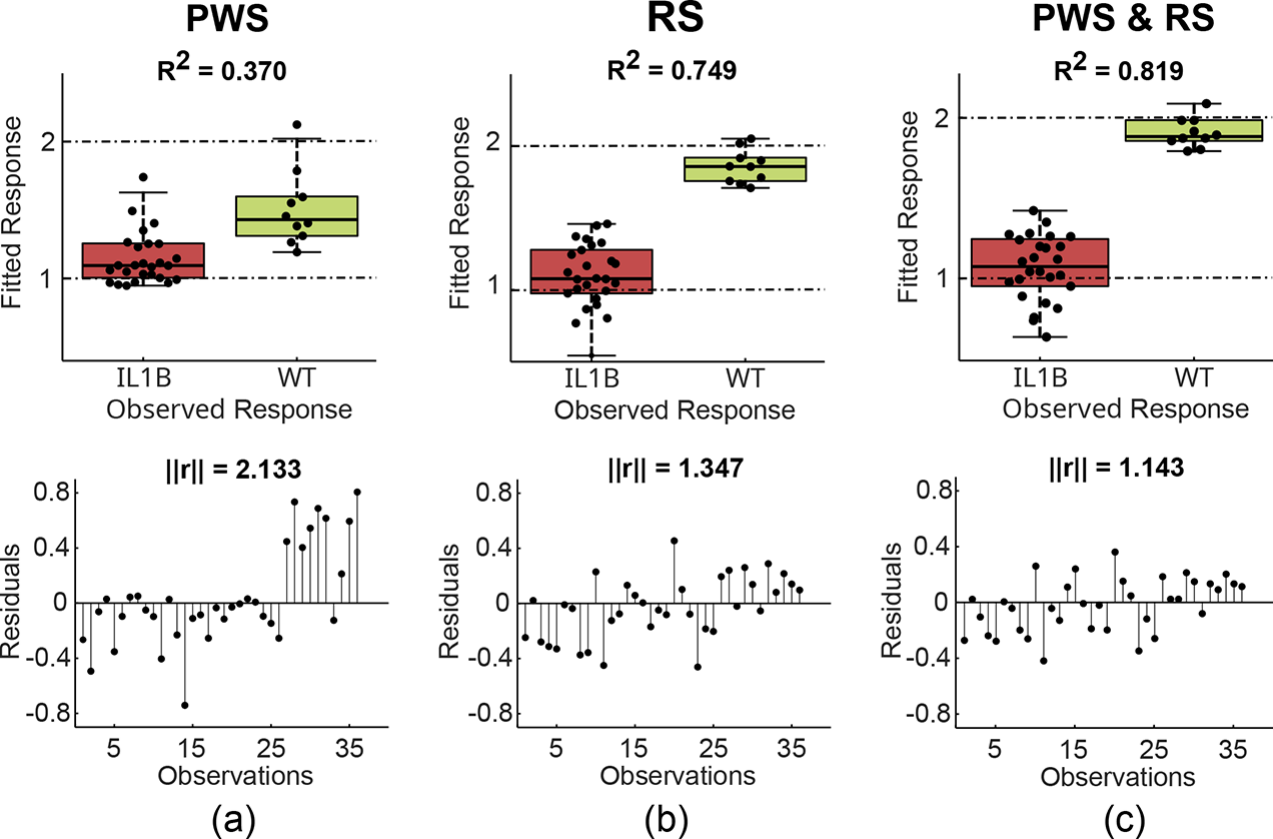
Classification results from the application of the partial least-squares discriminant analysis (PLS-DA) algorithm on data sets from IL1B forestomachs compared to wild-type (WT) data. (a) The fitted response (top) and the residuals as a function of observations (bottom) from the partial wave spectroscopy (PWS) measurements. (b) The fitted response (top) and the residuals as a function of observations (bottom) from the Raman spectroscopy (RS) measurements. (c) The fitted response (top) and the residuals as a function of observations (bottom) when PWS and RS measurements are combined. The algorithm performance is evaluated by means of *R* ^2^ and the norm of residuals (||*r*||) for each data set.

**Figure 6:**
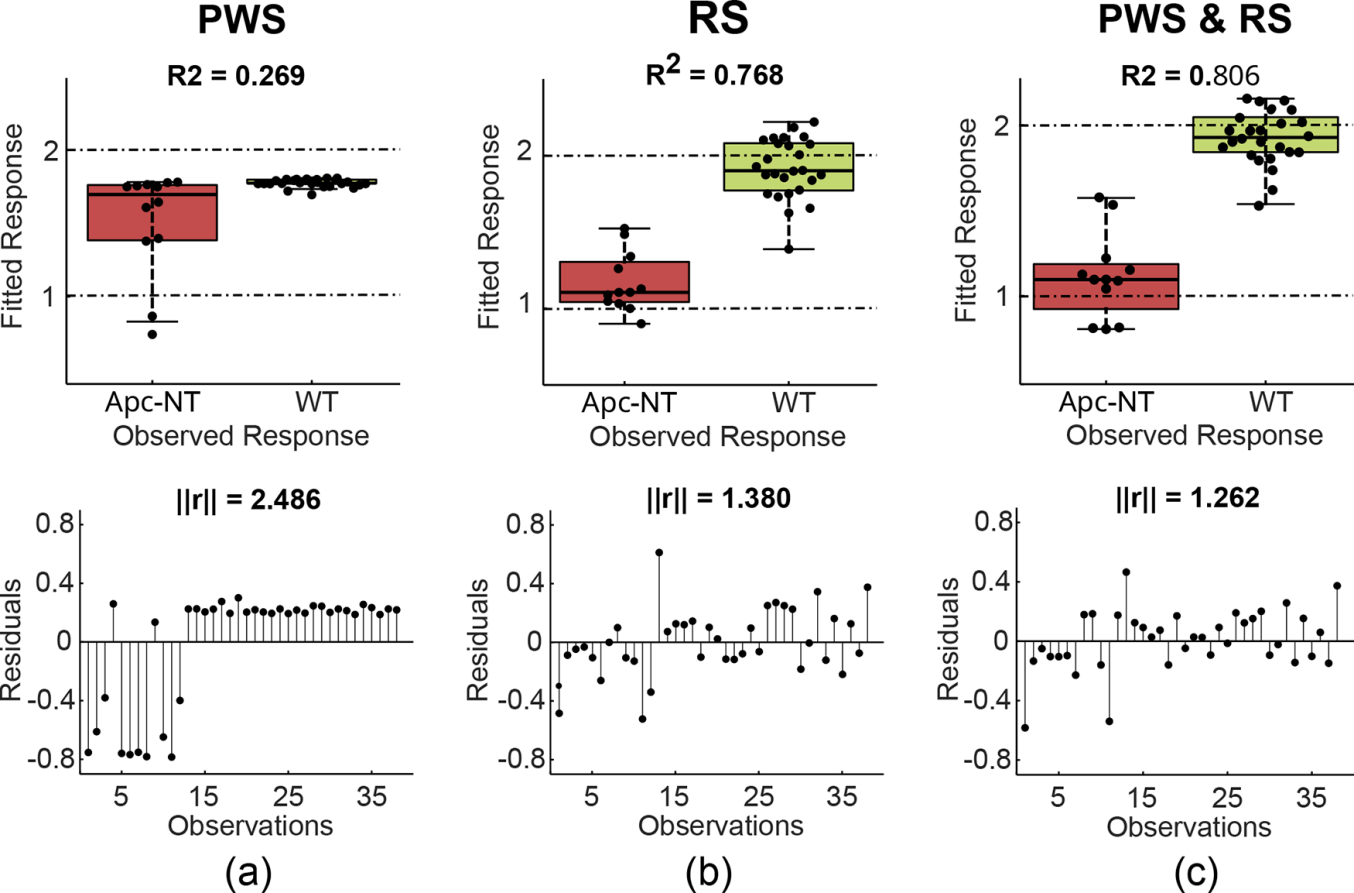
Classification results from the application of the partial least-squares discriminant analysis (PLS-DA) algorithm on data sets from Apc-NT intestines compared to control data. (a) The fitted response (top) and the residuals as a function of observations (bottom) from the partial wave spectroscopy (PWS) measurements. (b) The fitted response (top) and the residuals as a function of observations (bottom) from the Raman spectroscopy (RS); (c) the fitted response (top) and the residuals as a function of observations (bottom) when PWS and RS measurements are combined. The algorithm performance is evaluated by means of *R* ^2^ and the norm of residuals (||*r*||) for each data set.

## Discussion

The field cancerization concept implies that tumor-adjacent tissue shares similarities with the tumor itself, which are not yet histologically apparent.^1^ According to that, the molecular differences that appear between tumor and healthy tissue should also be found in the comparison between tumor-adjacent and healthy tissue, as well. When comparing ‘normal’ adjacent to precancerous tissue and healthy controls, we show statistically significant differences in Raman spectra that are in agreement with the RNA-seq analysis and histopathological findings. We also note that FC can present different molecular and optical profiles in different tissues and cancer types.

To investigate FC alterations in upper GI tract tissues, we used a clinically relevant transgenic animal model (L2-IL1B, ref as IL1B) that closely recapitulates human tumorigenesis through the metaplasia–dysplasia–adenocarcinoma sequence triggered by IL1B-driven chronic esophagitis.^28^ A higher concentration of amino acids is reflected in the intensities of the RS peaks of IL1B mice (Figure 2e). This is expected, as the FC concept^6^ states that disease progression is likely to be associated with inflammatory processes in tissue adjacent to premalignant tissue, such as forestomach tissue adjacent to premalignant SCJ tissue. Proliferation of proteins and amino acids in the diseased tissue is also in agreement with previous studies.^33^

Additionally, lower relative intensities for tissues adjacent to precancerous tumors in IL1B mice (Figure 2d) indicated decreased concentrations of lipids, phospholipids, and/or fatty acids in the inflamed tissues. This loss of lipids in the diseased tissue appears to be a commonly observed characteristic, not only in previous GI cancer studies^47^ but also in inflammatory activity assessments of ulcerative colitis (UC)^48^ and studies of FC in oral cancer.^34^ Moreover, decreased Raman signals associated with lipids were reported to be potentially related to eosinophilic inflammation and basal zone hyperplasia.^49^ The prevailing presence of carotene peaks in the WT group without chronic esophagitis is likely due to the anti-inflammatory or chemopreventive effect of carotenoids^50^ in WT tissues (Figure 2f). The anticarcinogenic properties of carotenoids have been studied using RS since Puppels’ seminal work in 1993.^51^

The IDM textural feature evaluated via GLCM^37^ shows higher mean values for IL1B forestomach samples in comparison to controls (Figure 2b), which can be correlated to morphological changes of the inflamed tissue. However, the resulting texture statistics for both groups have relatively high standard deviations (Figure S1a), suggesting tissue heterogeneity within the groups. In the case of IL1B tissues, this heterogeneity is expected, as inflammation levels varied between forestomach samples, which is also observed in the RS and RNA-seq results (Figure S1b,c). In WT tissues, the heterogeneity of the scattering measurements could be associated with the anatomy of the tissue samples, as the SCJ is very thin in WT mice (Figure 1a), and FOVs for scattering measurements could have partially included the SCJ tissue bordering the location of interest. To our knowledge, this is the first study of structural changes in inflamed forestomach tissue in the context of esophageal FC. Although the PWS structural differences detected in our study were not statistically significant, they are in agreement with independent studies probing FC-related changes in the upper GI tissue structure.^16,19,20^ Therefore, while the reflectance light scattering methods are capable of detecting subtle FC-related changes in upper GI tissue structure, their diagnostic accuracy still suffers due to the methods’ low specificity.

FC biomarkers of intestinal tumors were also examined in tissues from Apc mice. In this model, intestinal cancerogenesis is not driven by inflammation as in IL1B mice but by the knockout of the Apc gene.^29^ The focus was on detecting molecular and structural changes in regions surrounding premalignant tumors (Apc-NT). The histopathological evaluation confirmed that there were no cancerous alterations visible in the nontumorous regions (Apc-NT) of the Apc mice. The significant statistical differences (Figure 4d) observed at the 1620 cm^–1^ Raman band are likely associated with angiogenesis processes in tissues adjacent to tumors.^52^ The significant differences (Figure 4d) observed in the bands at 854, 879, and 1588 cm^–1^ imply an increase of amino acids and protein content in the tissues of the Apc-NT group, similar to the trend observed in IL1B samples. The consistency of biochemical alterations provides evidence that Raman spectral features reflect cancer-progression-related processes in FC tissues. At the same time, the difference associated with protein and lipid content (Figure 4d) has previously been reported as an indicator of disease progression status between tumor-adjacent tissue and tumors.^25^ Furthermore, we demonstrate that RS measurements correlate well with transcriptomic (RNA-seq) analysis. Overall, the distinctive differences in Raman spectra confirm the capability and utility of RS for detecting FC changes related to cancer development in tissues of an intestinal tumor mouse model driven by Apc knockout.

Similar to our observations in gastroesophageal tumor mouse models, the extracted IDM textural feature (Figure 4b) showed higher mean values for Apc-NT samples in comparison with controls, suggesting morphological changes of the affected normal-appearing tissue adjacent to tumors in Apc mice. Interestingly, in the case of the intestinal samples, texture statistics of the control group are consistent, with a very low standard deviation (Figure 4b and Figure S2a) compared to the gastroesophageal mouse model (both groups: WT and IL1B, Figures 2b and S1a) and the Apc-NT group (Figures 4b and S2a). These results reflect the similarity of the control group intestinal tissue samples and the consistent tissue structure throughout the intestines of healthy mice in comparison with the forestomachs. At the same time, high standard deviations in IDM values in the Apc-NT group imply high variation within the diseased tissue samples, which can be correlated to the carcinogenesis process, as well as to the location of the excised tissues (i.e., closer or farther from premalignant intestine tumors).

According to a previous study of colorectal field carcinogenesis,^17^ nanoscale changes are an early stage event in carcinogenesis, which is in agreement with our PWS data between Apc-NT and control groups. As expected, the observed differences are also evident when comparing Apc-T and control groups (Figure S5a,b). However, differences in PWS data between the Apc-T and Apc-NT groups (Figure S5c,d) are minor. Similar findings were previously reported in cells from colon cancer patients^53^ and intestinal neoplasia (MIN) mice,^54^ supporting the hypothesis that morphological changes occur early in lower GI carcinogenesis.

The classification of the obtained data (i.e., PWS image texture statistics and RS data) shows that the RS modality performed better than the PWS modality when employed separately (panels (a) and (b) in Figures 5 and 6). However, the classification metrics improved when PWS and RS data were combined (panel (c) in Figures 5 and 6), suggesting that joint structural and chemical information offers a performance advantage in FC probing.

It is important to highlight that a study conducted through ex vivo experiments with mice models may not precisely convey in vivo parameters such as those that occur during clinical procedures. Nevertheless, the biomolecular and structural changes detected are inherently similar to FC in human organs, since genetically engineered mouse cancer models faithfully reproduce human disease and can be temporally controlled and studied.

One of the limitations of the current study is the morphological gradient of the developing disease, a challenge that has been previously discussed in studies examining early detection in GI cancers.^55^ These studies pinpoint that disease-related changes are not uniform across a given sample, and therefore, measurements are likely to contain different tissue types (in terms of disease state) within one FOV. This limitation is evident in the relatively small contribution of PWS data to the overall results and PLS-DA classification. These misclassifications may stem from nonuniform changes across a given sample due to the spatial heterogeneity of the disease or possibly from probing at the periphery of more advanced microtumors. Consequently, measurements may include normal tissue regions that are indistinguishable between samples, particularly in the PWS mode, which features a larger FOV. Additionally, histopathology and RNA-sequencing analyses confirmed the absence of pathological changes in some samples from the disease-affected groups.

Another limitation is that we chose for our measurements forestomach tissue, which is not present in humans. However, since the forestomach presents squamous epithelium and is immediately next to the precancerous SCJ, as is the human esophagus,^6^ we believe that our measurements in the forestomach can reliably reflect FC observed in the human esophagus.

Despite these limitations, our study demonstrates that the RS-PWS system can detect functional changes related to cancer progression in gastroesophageal and intestinal tumor mouse models, even before visible changes occur. Moreover, the data obtained from optical measurements provide good agreement with RNA-seq findings. Further studies are essential to better understand the relationships between morphological and biochemical changes identified by optical measurements and important events in FC initiation and progression.

## Conclusion

In the current work, we used a hybrid RS-PWS microscope to examine early tissue alterations associated with FC by interrogating macroscopically normal tissues in FC-associated areas of fresh tissue samples from mice tumor models. We show statistically significant differences in Raman spectral bands and variations in PWS parameters of precancerous tissues from gastroesophageal and intestinal tumor mouse models compared with those of healthy controls. Our results are supported by RNA-seq analysis. Moreover, we demonstrate that by applying PLS-DA, combined data from RS and PWS can be used to distinguish between FC areas and areas in healthy controls more reliably than either individual modality. Overall, the distinctive differences in Raman spectra between macroscopically normal tissue located in FC zones and healthy control gastroesophageal and intestinal mouse tissue corroborate the potential of RS-PWS endoscopy for in vivo endoscopic examinations of FC.

## Supplementary Material















## Notes

Principal component analysis (PCA) and score plots for data acquired with the RS-PWS system and on RNA-Seq data (IL1B vs WT groups; Apc-NT vs control groups); optical measurement results (RS and PWS) for Apc-T samples; score plots; Raman spectra; boxplots; scatter plots; and data analysis results (PDF)
Sequencing metrics of the IL1B model (XLSX)
Sequencing metrics of the Apc model (XLSX)
DEGs tumor FS vs control FS (outliers are excluded) (XLSX)
DEGs APC-NT vs control (XLSX)
DEGs APC-NT vs APC-T (XLSX)
DEGs APC-T vs control (XLSX)

## §§

Bundesanstalt für Materialforschung and -prüfung (BAM), 12489 Berlin, Germany

## ∥∥

Clinical Radiology, Medical School OWL, Bielefeld University, Bielefeld, 33615, Germany.
