# Supplementary material for: Probing Field Cancerization in the Gastrointestinal Tract Using a Hybrid Raman and Partial Wave Spectroscopy Microscope
Source: Anal Chem. 2025 Jun 11;97(24):12642–53. doi: 10.1021/acs.analchem.5c00954 (PMC12199223; doi:10.1021/acs.analchem.5c00954)
Supplement: Supplementary file 1 [file ac5c00954_si_001.pdf]

Supporting information for "*Probing field cancerization in the gastrointestinal tract using a hybrid Raman and partial wave spectroscopy microscope*"

Elena Kriukova[a,b], Mikhail Mazurenka[a,b], Sabrina Marcazzan[a,b], Markus Tschurtschenthaler[c,d,e], Gerwin Puppels[f], Sarah Glasl[a,b], Dieter Saur[c,d,e], Moritz Jesinghaus[g,h], Marialena Pouliou[i], Marios Agelopoulos[i], Apostolos Klinakis[i], Michael Quante[j], Jorge Ripoll[k], Vasilis Ntziachristos[a,b,l], Dimitris Gorpas[a,b*]

[a] Chair of Biological Imaging, Central Institute for Translational Cancer Research (TranslaTUM), School of Medicine and Health & School of Computation, Information and Technology, Technical University of Munich, 81675 Munich, Germany
[b] Institute of Biological and Medical Imaging, Bioengineering Center, Helmholtz Zentrum München, 85764 Neuherberg, Germany
[c] Division of Translational Cancer Research, German Cancer Research Center (DKFZ) and German Cancer Consortium (DKTK), 69120 Heidelberg, Germany
[d] Chair of Translational Cancer Research and Institute of Experimental Cancer Therapy, Klinikum rechts der Isar, School of Medicine and Health, Technical University of Munich, 81675 Munich, Germany
[e] Center for Translational Cancer Research (TranslaTUM), School of Medicine and Health, Technical University of Munich, 81675 Munich, Germany
[f] RiverD International B.V., Marconistraat 16, 3029 AK Rotterdam, Netherlands
[g] Institute of Pathology, Technical University of Munich, 81675 Munich, Germany
[h] Institute of Pathology, University Hospital Marburg, 35043 Marburg, Germany
[i] Center for Basic Research, Biomedical Research Foundation of the Academy of Athens, 115 27 Athens, Greece
[j] Klinik für Innere Medizin II, Universitätsklinikum Freiburg, Freiburg, 79106, Germany
[k] Department of Bioengineering and Aerospace Engineering, Universidad Carlos III de Madrid, 28005 Madrid, Spain
[l] Munich Institute of Biomedical Engineering (MIBE), Technical University of Munich, 85748 Garching b. München, Germany

Corresponding author: Dimitris Gorpas, E-mail: dimitrios.gkorpas@tum.de

The file includes:

Principal component analysis (PCA) for data aquired with RS-PWS system and on RNA-Seq data (IL1B vs WT groups; Apc-NT vs Control groups)

RNA Sequencing: list of excel files

Supplementary Tables S1 to S6 (see the excel files)

Optical measurement results (RS and PWS) for Apc-T samples

Supplementary Figures S1 to S5

Principal component analysis (PCA) for data aquired with RS-PWS system and RNA-Seq data (IL1B vs WT groups; Apc-NT vs Control groups)

Principal component analysis (PCA) for optical measurements data

PCA was employed to visualize data obtained with the RS-PWS microscope by reducing the data dimensionality. As inputs for PCA, the normalized Raman spectra and values of the IDM textural feature were used. The scores of the first two components are shown using scatter plots. Visualizing the data as 2D scatter plots facilitates the identification of data clusters and outliers, and allows for the exploration of patterns in the dataset.

PCA for RNA-seq analysis

To assess the similarity between groups of samples, PCA was performed. Briefly, an x-D graph of the data (mice and genes) was first constructed and a correlation line added, which was generated using the sum squares method. This line comprises the eigenvector for PC1. Then, the proportion of variance that each PC accounts for was calculated by applying the following formula:

$$Var(PC) = \frac{SS(distance\ from\ the\ origin\ for\ PC)}{n-1}$$

A satisfying percentage of explained variance for selected PCs is about 80%. By combining the eigenvectors of the selected PCs, we can establish the score plot. The score plot is indicative of clusters, trends, and outliers. The built-in function plotPCA of the DESeq2 algorithm (Galaxy Version 2.11.40.6+galaxy2) for plotting PCA [1] on Galaxy was used and returned only the values for PC1 and PC2. PCA was conducted for distinct categories of samples (control vs 'normal'/tumor) of each model individually to visualize the patterns of clustering.

IL1B *vs WT groups*

To test whether differences in the IDM textural feature data imply slight changes in the tissue morphology of the IL1B mice compared to WT mice, we performed principal component analysis (PCA). The scatter plot of the first two principal component (PC) scores (Figure S1a) confirms both the difference between the groups and the high standard deviations of the IDM textural feature. PC scores are also widely spread. Thus, PWS measurements suggest the loss of a structured/well-organized texture in the forestomachs of IL1B mice, where both the mean IDM textural feature value and its standard deviation are higher.

Figure S1b shows the scatter plot of the first two PC scores for the RS data, indicating molecular differences between the healthy control WT group and the IL1B group.

Additionally, RNA-seq was conducted to molecularly characterize healthy and diseased tissue samples. PCA was then performed based on gene expression levels, and a score plot was constructed for all RNA-seq samples. The resulting plot (Figure S1c) indicates that the forestomachs of IL1B mice carry molecular profiles that readily discriminate them from healthy equivalents (WT), which is also in a good agreement with the PCA results from RS data (Figure S1b).

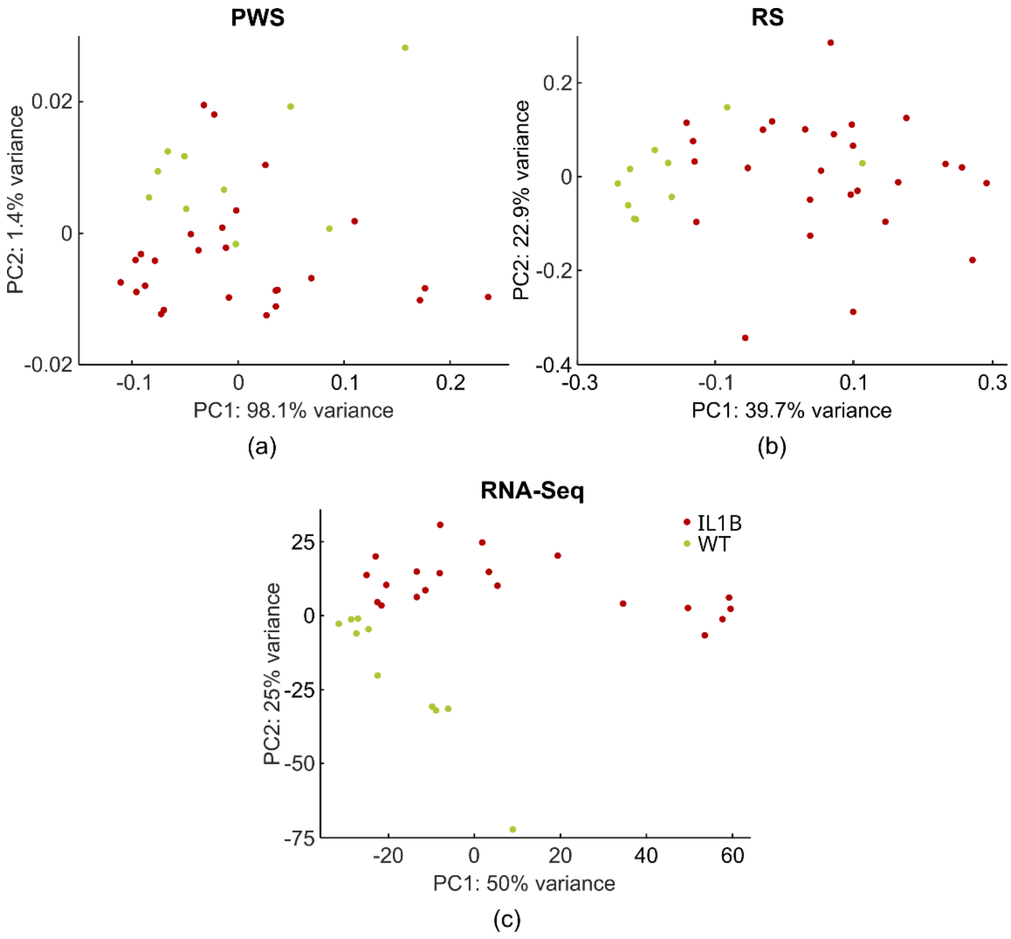

Figure S1. Score plots for the first two principal components (PCs) of (a) PWS data, (b) RS data, and (c) RNA-seq data for gastroesophageal tumor (IL1B) mouse model vs wild type (WT) groups.

Apc-*NT vs Control groups*

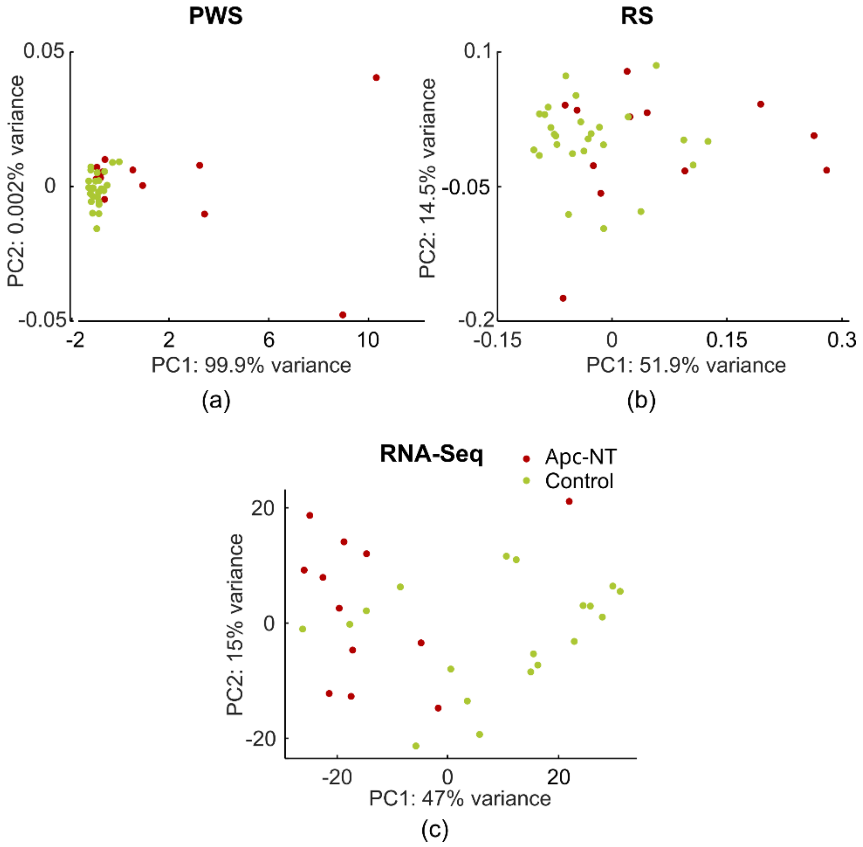

Figure S2. Score plots for the first two principal components (PCs) of (a) PWS data, (b) RS data, and (c) RNA-seq data for intestinal tumor (Apc-NT) mouse model vs Control (WT) groups.

Figure S2a confirms the observed variation of texture statistics in PWS images in the Apc-NT group, which is reflected in the score plot of the first two PCs. Here, the data points are widely spread compared to the control group data. Additionally, score plots of the first two PCs from RS and RNA-Seq data are shown in Figure S2b-c.

RNA Sequencing: list of XLSX files

1. Sequencing metrics (two files: ‘Supplementary_Table_S1_ Sequencing metrics_IL1B_model.xlsx’, ‘Supplementary_Table_S2_ Sequencing metrics_Apc_model.xlsx’; one for each cancer model). These files include transcript integrity number (TIN) scores, total reads, mapped reads, and uniquely mapped reads.

2. DEGs files. These files include normalized counts, Deseq2, biological processes (GO), KEGG pathways, and REACTOME for upregulated and downregulated genes.

1) For the IL1B model:
‘Supplementary_Table_S3_DEGs_Tumor_FS_VS_Control_FS_(outliers_are_excluded).xlsx’

2) For the Apc model: ‘Supplementary_Table_S4_ DEGs_APC-NT_vs_Control.xlsx’; ‘Supplementary_Table_S5_ DEGs_APC-NT_vs_APC-T.xlsx’; ‘Supplementary_Table_S6_ DEGs_APC-T_vs_Control.xlsx’;

In total, there are 6 excel files.

Optical measurement results (RS and PWS) for Apc-T samples

Considering that the comparison of Apc-NT vs Apc-T (Figure S3) is done between different tissue locations (normal and tumor) of the same mouse, molecular changes have a higher probability to be associated with tumor development. Statistical analysis of the Raman measurements yielded higher intensities ($p<0.01$, Figure S3b) involving free amino acids (proline, hydroxyproline, tyrosine – 830 cm^{-1}; ribose vibration, one of the distinct RNA modes – 974 cm^{-1}) and proteins (Amide I (a-helix) - 1658 cm^{-1}) in the NT group. The tumor tissue group had higher intensities ($p<0.05$, Figure S3c) of the following bands: 1093 cm^{-1} (symmetric phosphate stretching vibrations), 1320 cm^{-1} (DNA/RNA, CH deformation (proteins)) and 1340 cm^{-1} (nucleic acid modes). These spectral changes indicate differences in the nucleic acid and collagen content in

tissues which are consistent with DNA changes linked to pre-cancer and cancer transformation[2, 3]. Figures S3d and S3e show similar group distributions on the scatter plots of the first two PC scores resulting from the PCA of RS and RNA-seq data. Interestingly, two red data points, which appear in the middle of the Apc-T group (arrows, Figure S3d) are from mice with the highest number of macroscopically visible tumors. Therefore, differences detected by RS appear to be sensitive to disease progression.

The comparison of control (Control) vs tumor (Apc-T) groups yielded the highest number of statistically significant differences in Raman band intensities (Figure S4). Similar to the comparison with the Apc-NT group, tumor samples had higher intensities ($p<0.0001$, Figure S4c) than controls at the 1340 cm^{-1} band (nucleic acid modes) indicating differences in the nucleic acid and collagen content. Differences were also observed at 1578 cm^{-1} (guanine, adenine). Decreases in the intensities of bands in the Apc-T group (Figure S4d) involve free amino acids (830 cm^{-1}, proline, hydroxyproline, tyrosine), and proteins and lipids (1303, 1449, 1658 cm^{-1}, CH_3/CH_2 twisting or bending mode of lipid/collagen; C-H vibrations; Amide I). Peaks corresponding to stretching and free amino acids can be associated with cell adhesion, cell-matrix interaction, migration, catabolic processes and other processes which can be observed in both Control vs Apc-NT and Control vs Apc-T comparisons.

Furthermore, PCA for RNA-seq results of the Apc-T tissues show, as expected, nice separation between groups, indicating that tumors in Apc mice carry molecular changes that readily discriminate these tissues from healthy equivalents (Figure S4e). The same trend is also depicted in scatter plots of data from the Raman spectra of the respective groups (Figure S4e).

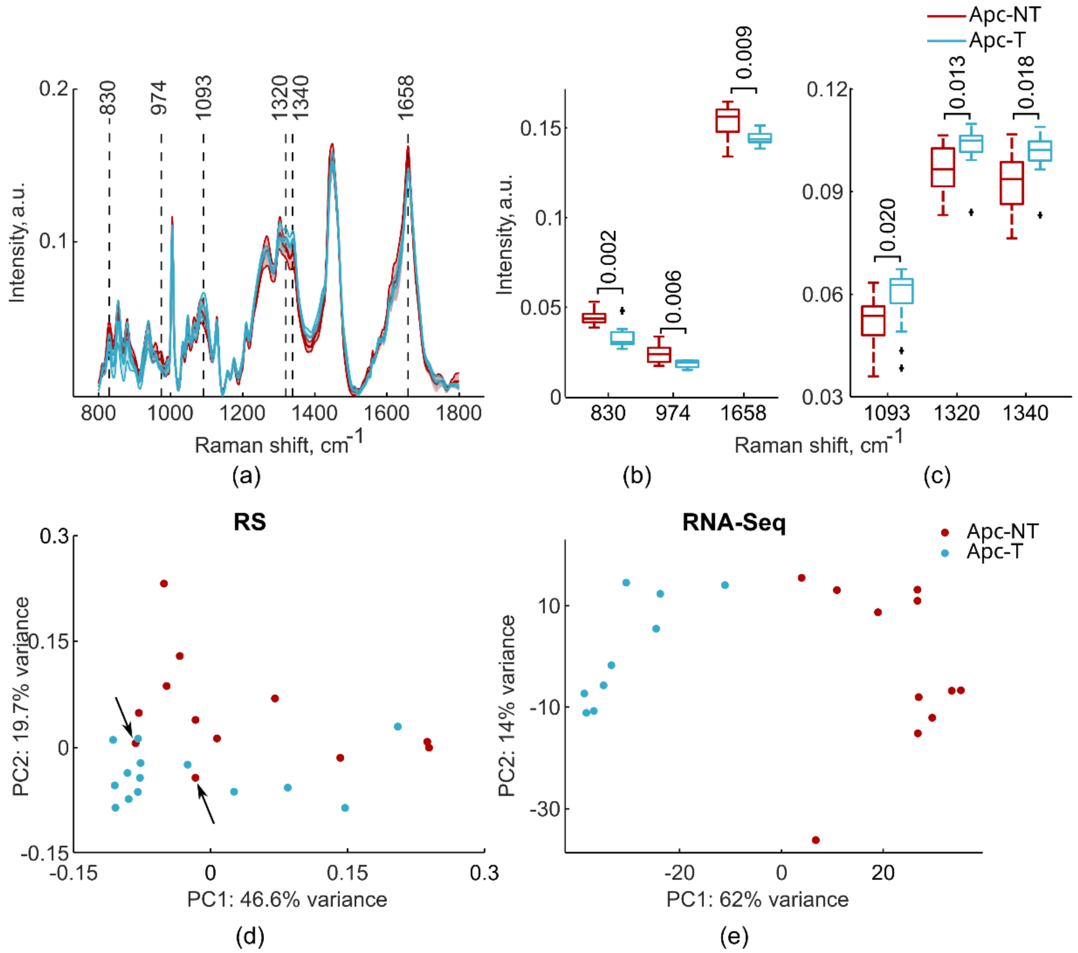

Figure.S3. Statistical analysis of the Raman measurements of macroscopically normal tumor-adjacent tissue samples (Apc-NT) compared to tumor samples (Apc-T). (a) Raman spectra of macroscopically normal intestinal tissue from Apc mice (Apc-NT, red) vs tumor tissue from Apc mice (Apc-T, blue) with mean and standard deviation indicated. (b-c) Boxplots of significantly different Raman spectra intensities (p-values are indicated within the bar graph; unpaired non-parametric Mann-Whitney U test). Scatter plots resulting from principal component analysis (PCA) of (d) Raman spectroscopy (RS) data (arrows indicate the data points from mice with the highest number of macroscopically visible tumors), and (e) RNA-sequencing (RNA-seq) data.

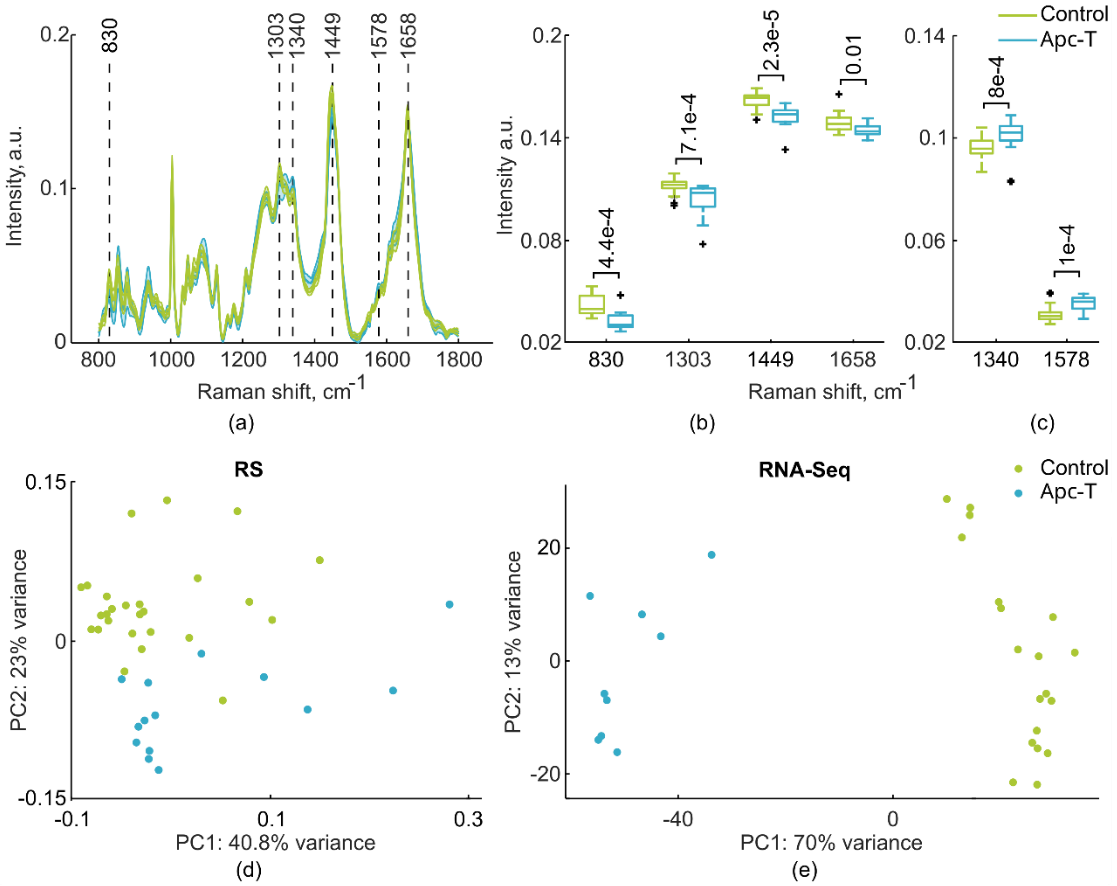

Figure. S4. Statistical analysis of the Raman measurements for tumor samples (Apc-T) compared to controls. (a) Raman spectra of the intestinal tumor tissue of Apc mice (Apc-T, blue) vs control mice (Control, green) with mean and standard deviation indicated. (b-c) Boxplots of significantly different Raman spectra intensities (p-values are indicated within the bar graph; unpaired non-parametric Mann-Whitney U test). Scatter plots resulting from the principal component analysis (PCA) of (d) Raman spectroscopy (RS) data, and (e) RNA-sequencing (RNA-seq) data.

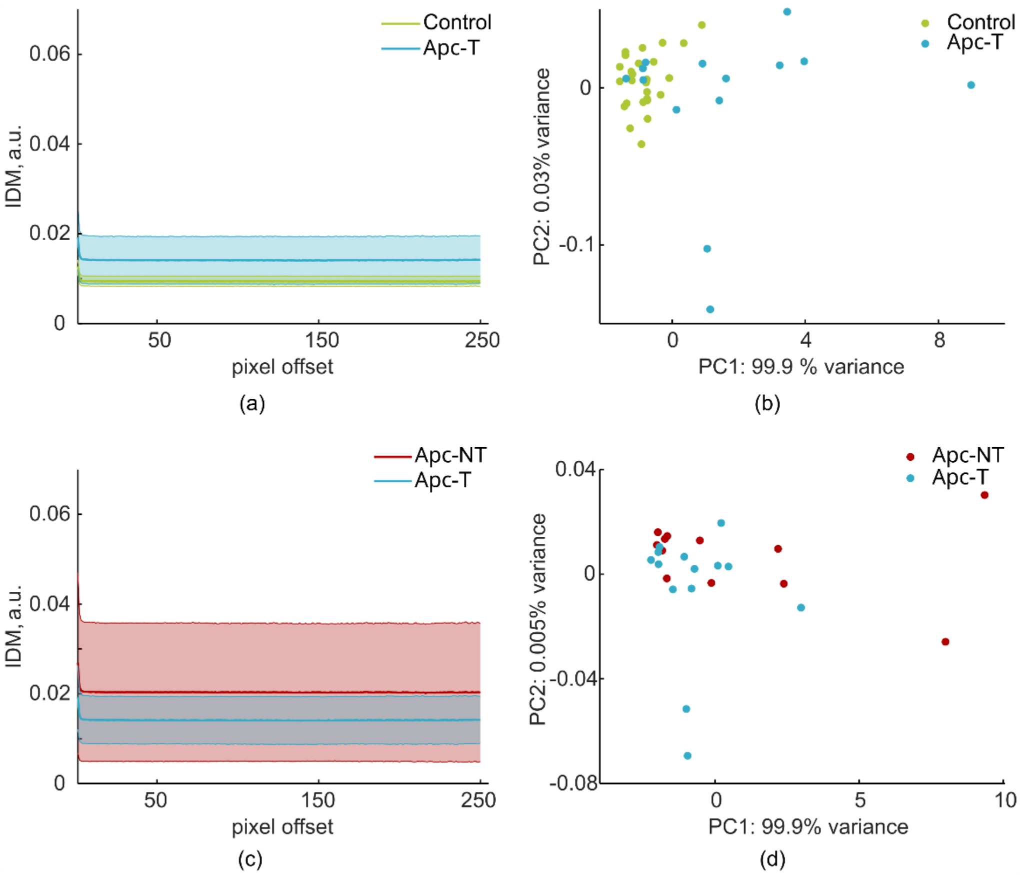

Figure. S5. Data analysis results from partial wave spectroscopy (PWS) measurements of tumor samples (Apc-T) compared to controls and to macroscopically normal, tumor-adjacent tissue samples (Apc-NT). (a, c) Distribution plot of the inverse difference moment (IDM) textural feature derived from Gray-level co-occurrence matrices (GLCM) calculated for each PWS image of intestine tissue samples from Apc tumor tissue (Apc-T, blue) vs intestinal tissue in wild type mice (Control, green) (a) and Apc tumor tissue (Apc-T, blue) vs macroscopically normal, tumor-adjacent tissue samples (Apc-NT, red) (c) with mean and standard deviation indicated; (b, d) Scatter plots resulting from principal component analysis (PCA) of PWS data, specifically Apc-T vs Control (b) and Apc-T vs Apc-NT (d).

(1) Love, M. I.; Huber, W.; Anders, S. Moderated estimation of fold change and dispersion for RNA-seq data with DESeq2. *Genome Biol* **2014**, *15* (12). DOI: 10.1186/s13059-014-0550-8.

(2) Bergholt, M. S.; Lin, K.; Wang, J.; Zheng, W.; Xu, H.; Huang, Q.; Ren, J. L.; Ho, K. Y.; Teh, M.; Srivastava, S.; et al. Simultaneous fingerprint and high-wavenumber fiber-optic Raman spectroscopy enhances real-time in vivo diagnosis of adenomatous polyps during colonoscopy. *J Biophotonics* **2016**, *9* (4), 333-342. DOI: 10.1002/jbio.201400141 From NLM Medline.

(3) Hiremath, G.; Locke, A.; Sivakumar, A.; Thomas, G.; Mahadevan-Jansen, A. Clinical translational application of Raman spectroscopy to advance Benchside biochemical characterization to bedside diagnosis of esophageal diseases. *J Gastroenterol Hepatol* **2019**, *34* (11), 1911-1921. DOI: 10.1111/jgh.14738.
